# Recycling of PBS and PBS Bio-Composites Containing Organic By-Product Waste

**DOI:** 10.3390/polym17192577

**Published:** 2025-09-24

**Authors:** Nadka Tz. Dintcheva, Giulia Infurna, Cristina Scolaro, Erika Alessia Di Liberto, Mariem Ltayef, Annamaria Visco

**Affiliations:** 1 Dipartimento di Ingegneria, Università degli Studi di Palermo, Viale Delle Scienze, Ed. 6, 90128 Palermo, Italy; giulia.infurna@unipa.it (G.I.); erikaalessia.diliberto@unipa.it (E.A.D.L.); 2 Dipartimento di Ingegneria, Università degli Studi di Messina, Contrada Di Dio, Villaggio S. Agata di Messina, 98165 Messina, Italy; cristina.scolaro@unime.it; 3 Laboratory of Asymmetric Synthesis and Molecular Engineering of Organic Materials for Organic Electron (LR18ES19), Faculty of Science, University of Monastir, Monastir 5019, Tunisia; ltayefmeriem@gmail.com

**Keywords:** polybutylene succinate (PBS), Beer Spent Grain Filler (BSGF), organic by-products, recycling, reprocessing, characterizations

## Abstract

The current work is driven by applying circular principles, and it investigated the potential recyclability of polybutylene succinate (PBS) containing brewer’s spent grain filler (BSGF, 30 wt%) in comparison to the recyclability of pure PBS. PBS is much more stable than the PBS/BSGF composite during processing cycles. Typically, thermomechanical degradation induces radical formation and branching of the macromolecular chain in PBS. Furthermore, PBS becomes less hydrophilic (by 53%, reaching 84°, approaching the 90° threshold), and its surface roughness increases by about 38% after five processing cycles. BSGF increases the viscosity of the melt, especially at low frequencies, and stabilizes the melt in the PBS/BSGF, which has lower torque variations during processing compared to pure PBS. Furthermore, BSGF in r-PBS/BSGF increases both hydrophilicity (by about 15%, from 75° to 64°) and surface roughness (by about 17%) after five processing cycles of the solid bio-composite and limits the formation of carboxylic groups during thermomechanical degradation. PBS is recyclable five times because it maintains its properties unchanged during extrusion cycles. At least two reprocessing steps are required for PBS/BSGF to obtain an optimal dispersion of BSGF, which can be re-extruded approximately three times. PBS/BSGF after four and five extrusion steps shows increased rigidity (E_t_ PBS/BSGF > E_t_ PBS) and reduced ductility (ε_b_ PBS/BSGF < ε_b_t PBS), which could limit the recyclability of the PBS-based composite.

## 1. Introduction

Polybutylene succinate (PBS) is a bio-based polymer representing a promising sustainable alternative to petroleum-based polymers. PBS has experienced significant growth in demand in recent years due to its sustainability and biodegradability potential. As is known, PBS is an aliphatic polyester produced from bio-based sources. It has well-balanced properties and high processability, like polypropylene, and has excellent biodegradability. Specifically, PBS has various physical and chemical properties like some synthetic polymers, such as high mechanical resistance, high melting temperatures, excellent chemical resistance, a good gas barrier, and antistatic properties. PBS is also known to be completely biodegradable by microorganisms in the natural environment [1,2,3].

However, PBS is known to be susceptible to thermo-oxidative degradation during processing and extrusion cycling [4]. Georgousopoulou et al. [5] studied the thermomechanical degradation of PBS, subjecting the material to five consecutive extrusion cycles. They highlighted its good recyclability because PBS, unlike other aliphatic polyesters (e.g., PLA and PHBV), degrades through branching/recombination reactions with a radical mechanism [6,7]. Therefore, the viscosity of the melt, the weight average molecular weight, and the polydispersity index increased. The concentrations of the terminal carboxylic group remained constant. The extent of final branching depends on the reprocessing temperature: increasing the latter resulted in earlier and more severe branching, while storing the resin in an environment with RH of 50% did not significantly interfere with the degradation pattern. If commercial antioxidants (Irganox^®^ 1010 (I1010) and Irgafos^®^ 168 (I168) by BASF SE Ludwigshafen (Germany) are added to PBS in amounts of 0.1 and 0.5% (*w*/*w*)), it can be stabilized profitably.

A natural antioxidant that can stabilize PBS of biological origin are polyphenols contained in wine grape pomace (WP, up to 20 wt%), studied by Hiller et al. [4]. The authors observed that WP is an efficient stabilizer for PBS, at low cost, and of biological origin. The characteristic thermal properties of the materials are almost unchanged, and the mechanical properties have been altered within the expected limits.

Nanni et al. [8] evaluated the possibility of using a laboratory-produced grape marc extract (GPext) and a commercial grape seed tannin extract (T) as natural PBS stabilizers, through thermomechanical degradation tests (reprocessing) and thermo-oxidation (oven ageing). GPext was not efficient because it is made of oligomeric polyphenols and could have undergone autoxidation, releasing peroxides and therefore new radicals, capable of accelerating the degradation of PBS. Instead, additive T resulted in the molecular weight of PBS remaining unchanged both after six extrusion cycles and after 300 h of oven ageing and increasing the degradation temperature by more than 20 °C. The efficiency of T is related to its long-chain polyphenolic structure that functions as a chain extender between broken polymer chains through inter- and intra-molecular hydrogen bonds, postponing the effects of degradation.

Furthermore, due to its sustainable nature, PBS is considered a suitable biopolymer matrix for the formulation of biocomposites aimed at advanced applications in sectors such as packaging, agriculture, and biomedicine. Mochane et al. [9] reviewed the development of PBS-based composites reinforced with natural filler derived from areca, banana, bamboo, hemp, jute, kenaf, palm, pineapple, and sisal, noting that a key challenge lies in the weak adhesion between hydrophobic PBS and hydrophilic natural fillers. This limitation can be addressed through surface treatments such as alkali modification and the use of coupling agents. In addition, filler type, content, and processing methods significantly influence mechanical performance and moisture resistance, supporting the potential of PBS-based biocomposites in sectors such as automotive, construction, agriculture, and packaging due to their biodegradability and low weight.

Considering the principles of materials circularity and sustainability, the formulation of biocomposites using organic residues is a key strategy for the valorisation of by-product waste. Brewer’s spent grain (BSG), the most abundant by-product of beer brewing—with approximately 20 kg generated per 100 L of beer [10]—is produced globally at an estimated 40 million tons per year and is primarily used for animal feed, biogas production, or composting [11,12]. BSG is a heterogeneous lignocellulosic material rich in proteins (20–36%), fibre (30–70%), lipids (4.5–8.3%), and ash (2.5–3.3%). Due to its composition, BSG has been investigated for its antioxidant and antimicrobial properties, with particular attention to how extraction conditions affect its functionality. Notably, Hadinoto et al. [13] demonstrated that high alkaline pH (11–12) improves protein extraction yield and enhances emulsifying capacity by promoting a more balanced amino acid profile and increased protein unfolding. Therefore, BSGF can be considered a suitable filler for bioplastics due to its availability, low cost, eco-friendliness, and waste-based nature.

Inspired by the idea of applying the principles of material circularity and sustainability, and considering the possibility of formulating, using, and recycling PBS-based biocomposites, this work investigates the re-processability of polybutylene succinate (PBS) containing brewer’s spent grain filler (BSGF), also compared to that of pure PBS. Specifically, to simulate the extrusion cycling, PBS and PBS/BSGF were reprocessed by extrusion up to five times, and the materials were subjected to detailed characterization after each reprocessing step. Rheological, morphological, and physic-mechanical tests have been performed on pure PBS and on the PBS/BSGF composite during five extrusion steps. The tests highlighted how BSGF changes rheological behaviour during the processing in the extruder, thereby changing the physical-mechanical features of the resulting recycled blends. To the best of our knowledge, the recycling of these blends PBS/BSGF has not been investigated so far. Therefore, this study advances existing knowledge by highlighting that PBS-based composites are only recyclable up to three extrusion steps, whereas the recyclability of PBS remains unchanged up to five extrusion steps.

## 2. Materials and Methods

### 2.1. Materials

Commercial PolyButylene Succinate (PBS, grade TH803S, lot TH803S-Bio D 202205262405A2, produced in 2022), has been purchased by Xinjiang Blue Ridge Tunhe Sci.&Tech. Co (Beijing Nan Lu, Changji city, Xinjiang, China) and tested in July 2024.

To obtain a usable filler, the BSG was appropriately transformed through a manufacturing process with the automated plant for the treatment of agro-food waste MTP-Mechanical treatment Prototype at the University of Messina, which includes drying at 80 °C overnight/milling/sieving to obtain the spent grains of brewer’s grain (BSGF) with a granulometry lower than 100 µm. BSGF has been introduced in PBS at 30 wt%, according to a previous investigation that revealed the effectiveness of this amount [14].

### 2.2. Reprocessing PBS and PBS/BSGF

In order to investigate the recyclability of PBS and the PBS/BSGF composite, PBS and BSGF were first dried overnight in a vacuum oven at 80 °C. For the composite, the materials were pre-mixed in the solid state and subsequently processed using a co-rotating twin-screw extruder (model EBV 19/35 D, OMC, Turin, Italy) with a temperature profile of 140–145–150–150–150–150 °C.

To simulate recycling, the materials were reprocessed under the same temperature conditions. However, it was necessary to optimize the operating parameters by maintaining a constant feed rate (3.5 ± 0.2 × 10^−4^ kg/s) while adjusting the screw rotation speed across the extrusion cycles. Specifically, screw speeds of 30, 50, 70, 80, and 95 rpm were applied from the first to the fifth cycle, respectively. The changes in the screw speeds were necessary in order to maintain a constant flow rate. Moreover, once steady-state conditions were achieved, the torque was recorded as a function of time throughout the extrusion process.

Throughout this work, the recycled neat PBS material is referred to as r-PBS, while materials corresponding to specific extrusion cycles are denoted as r-PBS_n (with n = 1–5). The same notation is adopted for the composites, referred to as r-PBS/BSGF and r-PBS/BSGF_n, respectively (with n = 1–5).

### 2.3. Characterisations

#### 2.3.1. Rheological Analysis

Rheological tests were performed using a strain-controlled rheometer (mod. ARES G2 from TA Instrument, New Castle, DE, USA) in parallel plate geometry (plate diameter 25 mm). The complex viscosity (η*) and storage (G’) and loss (G”) moduli were measured by performing frequency scans from ω = 10^−2^ to 10^2^ rad/s at the same processing temperatures (150 °C). The strain amplitude was γ = 5%, which preliminary strain sweep experiments showed to be low enough to be in the linear viscoelastic regime.

#### 2.3.2. Melt Flow Index Analysis

Melt flow index tests were performed using the Instron MFi5 machine (ITW Test And Measurement Italia S.r.l.—Instron CEAST Division, Pianezza (TO), Italy) according to the ASTM D1238-23 standards at 190 °C with the fixed nominal load of 2.16 kg [15]. The data presented are the average values from three measurements for each type of sample.

#### 2.3.3. Morphological Analysis

The microstructure of the samples was investigated using a Scanning Electron Microscope (SEM) imaging, carried out with a Phenom Pro X instrument (Thermo Fisher Scientific, Waltham, MA, USA). Prior to SEM analysis, samples were fractured in liquid nitrogen. The fractured surface of each sample was sputtered (Scancoat Six Edwards, Crawley, UK), with a thin layer of gold under argon atmosphere for 90 s, in order to avoid electrostatic charging under the electron beam.

#### 2.3.4. ATR-FTIR

To examine the chemical structures of r-PBS and r-PBS/BSGF as a function of the extrusion cycle, attenuated total reflectance Fourier transform infrared (ATR-FTIR) spectroscopy was performed using a Perkin-Elmer Spotlight 200i FTIR Microscopy System (710 Bridgeport Avenue, Shelton, CT06484, US) with Spectrum 3. A wavelength ranging from 600 to 4000 cm^−1^ was used at a resolution of 4 cm^−1^, with 32 scans. Hydroxyl and carbonyl indices were calculated using the following equations:

(1) $$Carbonyl\;Index\left(C.I.\right)=\frac{A_{1710}}{A_{2920}}$$

(2) $$Hydroxyl\;Index\left(C.I.\right)=\frac{A_{3600-3200}}{A_{2920}}$$

where *A* represents the peak area. According to the literature [5,16], the peak at 2920 cm^−1^ was chosen as the reference peak in the present study because it exhibits the least variation during the weathering.

#### 2.3.5. Roughness Measurements

The surface roughness (Ra, see Equation (3)) was calculated by the portable and compact roughness tester, Mitutoyo Surftest, mod. SJ-210-Series 178 (Mitutoyo S.r.l., Milan, Italy):

(3) $$Ra=\frac{1}{N}\sum_{i=1}^{n}\left|Yi\right|$$

where Ra is the arithmetic mean of the absolute values of the deviations of the evaluation profile (Yi) from the mean line. The data presented are the average values from ten measurements for each type of sample.

#### 2.3.6. Water Contact Angle Measurements (WCA)

The wettability of the materials was evaluated by the contact angle “theta” (*θ*) measurement (DMs-401, KYOWA, Milan, Italy), according to the international standard ASTM D7334 [17], by depositing 2 μL drop of deionised water on the horizontal surface of the sample. The wettability was derived from Equation [18,19]:

(4) $$\theta_{w}=2arctg\left(\frac{2h}{d}\right)$$

(5) $$\theta_{y}=arcos\left(\frac{{cos\theta }_{w}}{r}\right)$$

where *θw* is the Wenzel contact angle, *θy* is the Young contact angle (not dependent on the roughness), *d* is the diameter (in mm), and *h* is the height (in mm) of the drop. The data presented are the average values from ten measurements for each sample type.

#### 2.3.7. Hardness

Shore D hardness tests were performed with a PCE-HT210 durometer, offering digital display high precision offer a resolution of 0.2 hardness units [20]. The data presented are the average values from ten measurements for each type of sample.

#### 2.3.8. Tensile Test

The mechanical tensile tests were carried out with dog-bone specimens obtained according to the standard ASTM D638-14 [21] on a universal testing machine model Z005 (Zwick-Roell, Ulm, Germany) equipped with a 2.5 kN load cell. The specimens (lengths = 100 mm, widths = 10 mm, and thicknesses = ca. 1 mm) were tested at room temperature (20 °C) and RH of 43%, at a displacement speed of 2 mm/min. For each material, nine samples were tested. The average values of mechanical parameters, including elastic modulus (E_t_), tensile strength (σ_b_) and elongation at break (ε_b_), were calculated.

#### 2.3.9. Differential Scanning Calorimetry (DSC) Analysis

The degree of crystallinity of r-PBS and r-PBS/BSGF composites was determined by differential scanning calorimetry (DSC) TAQ500 instrument (TA Instruments, New Castle, DE, USA) according to Equation (6) [14]:

(6) $$\chi_{c}\left(\%\right)=\left(\frac{{\Delta H}_{m}}{{\Delta H}_{0\;}\;\times \;\left(1\;–\;w\right)}\right)\;\times \;100\;$$

where χ_c_ is the degree of crystallinity (%); ΔH_m_ is the melting enthalpy of the sample (J/g); ΔH_0_ is the melting enthalpy of PBS with 100% crystallinity PBS, and corresponds to 110.3 J/g [22]; and w is the weight fraction of BSGF in the composites.

#### 2.3.10. Statistical Analysis

The mean differences and standard deviations of wettability, roughness, hardness, and tensile test were calculated with Prism 8.0.2 statistical software (GraphPad, Inc., La Jolla, CA, USA). The Shapiro–Wilk test was used for normality and lognormality tests of data, and the Brown–Forsythe test was used for homogeneity of the variance test. Therefore, they were statistically analyzed by using two-way analysis of variance (ANOVA) and the Tukey post hoc test for multiple comparisons at a level of significance set at *p* < 0.05.

## 3. Results and Discussion

### 3.1. Processing of r-PBS and r-PBS/BSGF

**Figure 1:**
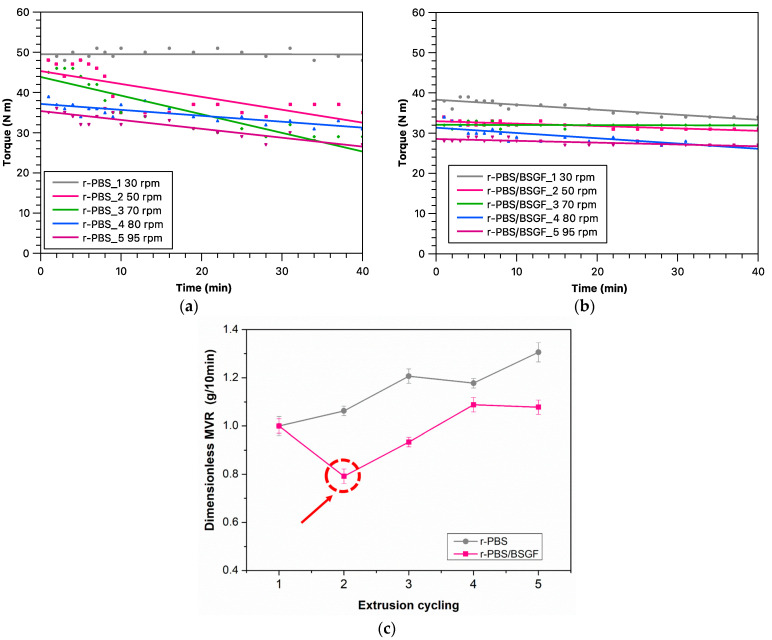
Torque trends of (**a**) r-PBS and (**b**) r-PBS/BSGF as a function of extrusion time and (**c**) dimensionless MFI of r-PBS and r-PBS/BSGF vs. extrusion cycling.

Figure 1a,b show the torque trends for r-PBS and r-PBS/BSGF (for both systems from the first to the fifth extrusion cycles) as a function of extrusion time. It should be noted that these torque trends are not related to the residence times of the processed materials, but they can be considered to be an approximate extent of the molten-state stability and processing capacity of the systems, keeping the extrusion flow rate unchanged. The torque trend of r-PBS_1 exhibits an almost constant value, corresponding to a negligible slope, suggesting that the material maintains processing stability over the entire extrusion duration considered (i.e., 40 min). It appears that the linear fit slopes of the torque trends for r-PBS_2-5 decrease as the processing steps increase, suggesting a reduction in molten-state stability. In order to maintain the extrusion flow rate unchanged, it was necessary to increase the screw speed about three times, i.e., from 30 rpm to 95 rpm, to ensure a constant flow rate for r-PBS, and this could be understood considering that during the extrusion cycling r-PBS_2-5 underwent thermo-mechanical degradation due to the mechanical stress and absorbed moisture (environmental humidity) during the reprocessing steps. Also, according to the literature, the presence of an organic filler, such as bran, and a plasticising oil in the biopolymer PLA/PBSA blend matrix causes a decrease in the torque during extrusion because of the ability of the filler to absorb humidity and the oil to slide the macromolecules, exerting both plasticising effects [23]. Therefore, the interpretation of the torque trends for r-PBS/BSGF turns out to be a much more complex phenomenon than for r-PBS because it must also be considered that both PBS and BSGF can absorb humidity during the extrusion cycling. It can be seen that the torque trend for r-PBS/BSGF_1 decreases slightly over the extrusion time considered. Furthermore, to keep the extrusion flow rate constant for both r-PBS and r-PBS/BSGF, the screw speed must be increased by more than three times, from 30 rpm to 95 rpm, even if the torque trends of bio-composites show fewer reductions in linear fit slopes during the extrusion time considered, suggesting that r-PBS/BSGF exhibit greater molten-state stability in the reprocessing extrusion steps than neat r-PBS. Although both PBS and BSGF can absorb humidity during extrusion cycling, the presence of BSGF exerts a beneficial effect on the molten-state stability.

The dimensionless melt flow Index (MFI) values vs. Extrusion cycle are reported in Figure 1c. Generally, the MFI values grow in both r-PBS and r-PBS-BSGF during the extrusion cycling. These data indicate that there is a 30.6% increase in melt flow in r-PBS from cycle one to cycle five, as can be expected from the structural variation (decrease in molecular weight and after the thermomechanical degradation of the processing). According to the torque data, the presence of BSGF has a helpful effect on the molten-state stability because the melt flow index increase is lower in r-PBS/BSGF from cycle one to cycle five (+7.8%) compared to PBS. As mentioned above, the MFI values increase with the extrusion cycles. However, in r-PBS/BSGF it is noted that at the second extrusion cycle, there is a decrease in MFI rather than an increase, contrary to the general trend (see red arrow in Figure 1c). This may be due to a better dispersion of the filler in the matrix, aided by the second extrusion.

### 3.2. Rheological Behaviour and Morphological Observations of r-PBS and r-PBS/BSGF

**Figure 2:**
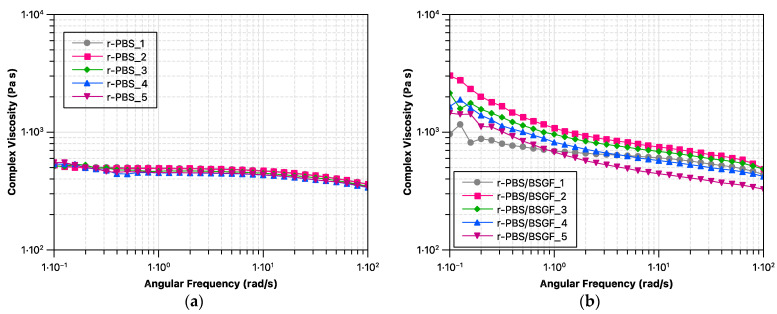
Complex viscosity of (**a**) r-PBS and (**b**) r-PBS/BSGF as a function of frequency.

**Figure 3:**
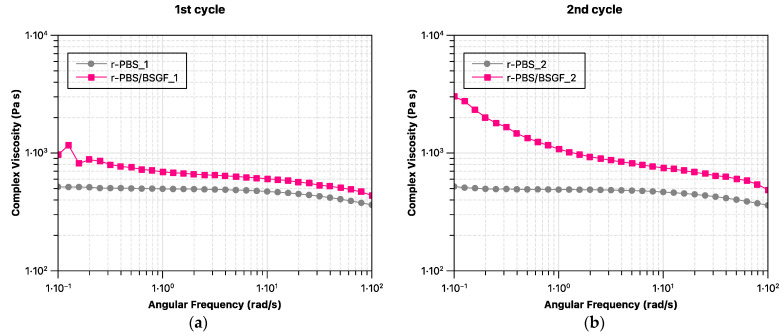

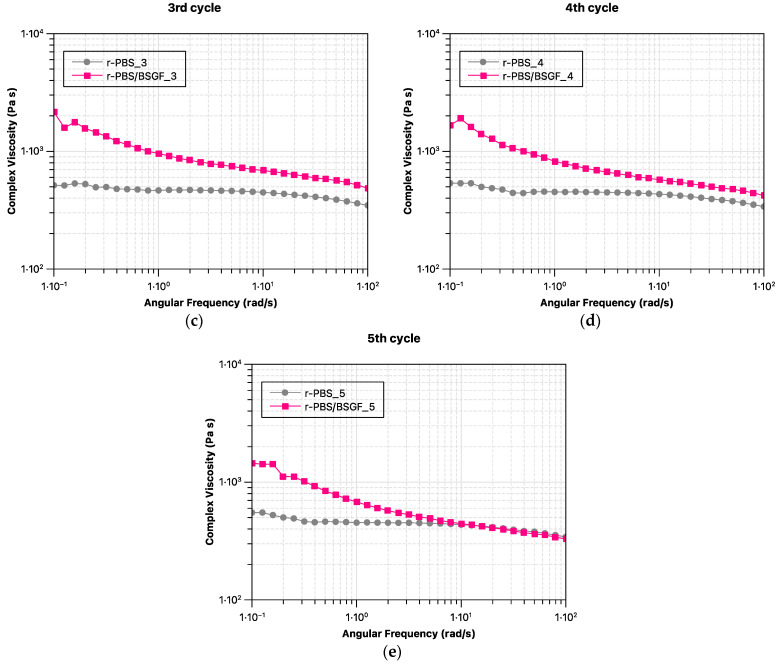
Comparison of viscosity trends of r-PBS and r-PBS/BSGF at different extrusion cycles: (**a**) first cycle, (**b**) second cycle, (**c**) third cycle, (**d**) fourth cycle, and (**e**) fifth cycle.

Figure 2a,b show the complex viscosity trends of r-PBS and r-PBS/BSGF as a function of frequency, and Figure 3a–e compare r-PBS and r-PBS/BSGF in the same reprocessing step. It can be seen that the rheological behaviour of the PBS remains unchanged when reprocessed by extrusion up to five times, as the viscosity trends slightly decrease, and as is noticeable, they are very close to each other. It appears that PBS has slightly lower viscosity values at the first and second extrusion cycles, and at the fourth and fifth extrusion cycles the viscosity curves appear lower compared to the others. The rheological behaviour of r-PBS from the first to the fifth extrusion step is the result of the balance between chain scission, which is caused by thermomechanical stress acting during the extrusion process, and chain branching. Clearly, at higher extrusion steps, thermomechanical stress has a greater effect than chain recombination.

Different viscosity trends are found for the r-PBS/BSGF bio-composite; the r-PBS/BSGF samples show well-pronounced yield stress at low frequencies and well-pronounced shear thinning at high frequencies due to the presence of BSGF. Interestingly, it appears that the first step is not sufficient to disperse the BSGF, as can be seen in Figure 3a. The difference in the viscosity trends of r-PBS/BSGF_2 and r-PBS_2, see Figure 3b, appears to be significant over the whole frequency range studied, and the latter could be attributed to the occurrence of a better BSGF dispersion during the second reprocessing step, according to the MFI results discussed before. It should be noted that the difference between r-PBS and r-PBS/BSGF decreases slightly from the third to the fifth reprocessing step, see Figure 3c–e. Therefore, at the fifth reprocessing step, the viscosity values for r-PBS/BSGF_5 and r-PBS_5 overlap in the high frequency range, indicating an equal processing capacity of both systems, and further, as expected, the presence of BSGF is detected at low frequency, resulting in the appearance of a well-visible yield stress.

Therefore, the rheological behaviour of r-PBS/BSGF samples is governed by the balance of the effects occurring in r-PBS and the presence of BSGF. As discussed above, the rheological behaviour of r-PBS depends on chain scission due to thermomechanical stress and/or chain branching resulting from random radical recombination. As expected, the presence of BSGF further complicates this behaviour. During the first two extrusion steps, the BSGF exerts a reinforcing action until it is optimally dispersed in the matrix. From the third to fifth extrusion steps, the well-dispersed BSGF can also prevent radical recombination and/or chain branching.

Although the detection of torque trends (Figure 1a,b), MFI (Figure 1c), and complex viscosity trends (Figure 2 and Figure 3) refer to the molten state of r-PBS and r-PBS/BSGF, these analyses detect two different behaviours; specifically, the torque and MFI trends are related to the molten-state stability during extrusions (keeping the extrusion flow rate constant), whereas the oscillatory tests also take into account the BSGF orientation obtained during the rheological test, and the latter could be considered responsible for the complex viscosity trends shown in Figure 2 and Figure 3.

Furthermore, according to the literature, PBS degrades upon thermo-mechanical stress, forming radicals and evolving to the formation of branched structures. It must be taken into account that the times (durations) of MFI analysis and the oscillatory test are different. Additionally, BSGF suppresses branching formation and hinders radical-mediated recombination by acting as a radical quencher or by physically restricting polymer chain mobility. When properly dispersed, BSGF contributes to mechanical reinforcement, improving melt viscosity retention and reducing chain scission propagation.

**Figure 4:**
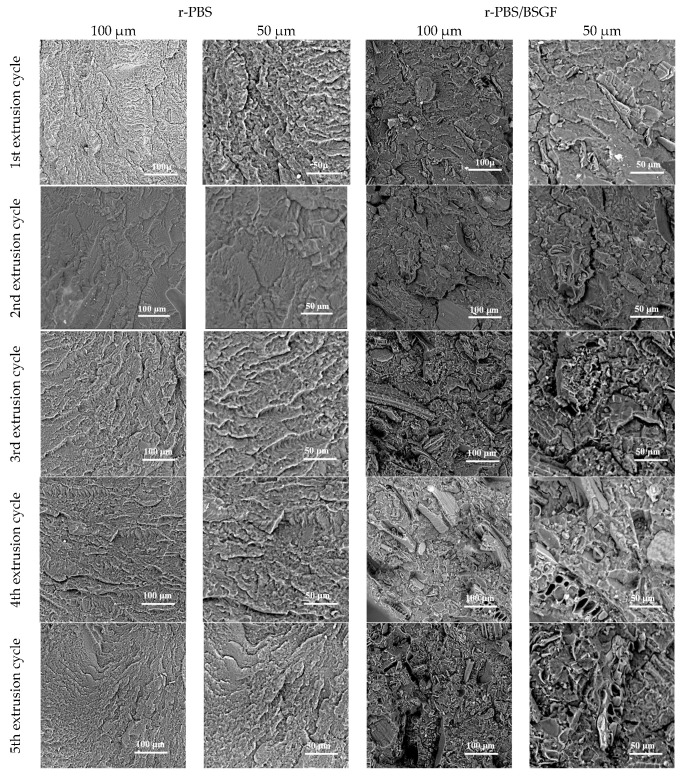
SEM images of r-PBS and r-PBS/BSGF at two different magnifications.

Figure 4 shows the SEM morphology observations at two different magnifications of the nitrogen fractured surfaces of r-PBS and r-PBS/BSGF. Consistent with the rheological behaviour, the morphologies of r-PBS do not show significant changes during extrusion cycling. It should be noted that the morphology of r-PBS_1 appears slightly irregular and remains almost unchanged for r-PBS_2-5. Furthermore, the high magnification observations show the flake morphology for r-PBS.

Interestingly, according to the rheological analysis, the morphologies of r-PBS/BSGF change as the extrusion cycling increases. In particular, both the appearance of the PBS host matrix and the dispersion of BSGF in the matrix change with extrusion cycling. It appears that the decoupling between PBS and BSGF and the uneven BSGF orientations become more visible with increasing processing steps. Furthermore, the morphologies of r-PBS/BSGF_4 and r-PBS/BSGF_5 show the presence of small voids, probably due to the occurrence of thermal degradation phenomena of cellulose–lignin structures. It should be noted that the r-PBS/BSGF_2 seems to have a better morphology in terms of less matrix degradation and better BSGF dispersion. The latter confirms the rheological trends and could be understood considering that only one reprocessing step is not sufficient to obtain a good BSGF dispersion in the PBS polar matrix. Therefore, at least two extrusion cycles are required to achieve a favourable balance between BSGF dispersion and matrix thermo-mechanical degradation.

### 3.3. Surface Analysis: Wettability, Spectroscopy and Roughness of r-PBS and r-PBS/BSGF

**Figure 5:**
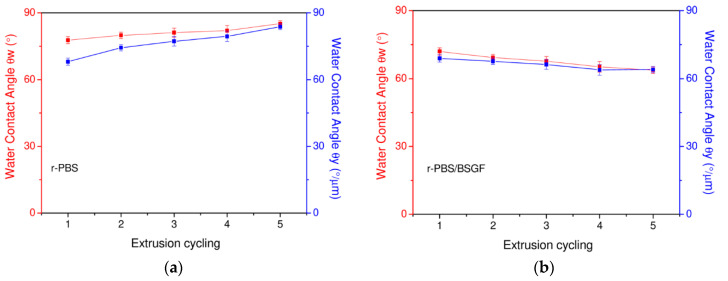
Water contact angle (WCA) values (**a**) *θ_w_* and (**b**) *θ_y_* for r-PBS (**left**) and r-PBS/BSGF (**right**) vs. extrusion cycling.

To verify the ability to absorb water/humidity/moisture of r-PBS and r-PBS/BSGF, the water contact angle (WCA) measurements are carried out, and the results obtained, which are extremely significant, are shown in Figure 5a,b. It is worth noting that r-PBS and r-PBS/BSGF show two different, opposite trends. In particular, for r-PBS both *θ_w_* (r-PBS_1 and r-PBS_5 are approximately 78° and 85°, respectively, *p* = 0.0008) and *θ_y_* (r-PBS_1 and r-PBS_5 are approximately 55° and 84°, respectively, *p* < 0.0001) values increase with increasing processing steps; PBS is therefore less hydrophilic after five processing cycles because *θ_y_* increases by about 53% reaching 84°, close to the 90° threshold.

In the bio-composite r-PBS/BSGF, both *θ_w_* (r-PBS/BSGF_1 and r-PBS/BSGF_5 are approximately 72° and 64°, respectively, *p* = 0.0001) and *θ_y_* (r-PBS/BSGF_1 and r-PBS/BSGF_5 are approximately 75° and 64°, respectively, *p* < 0.0001) values decrease with increasing processing steps. In detail, the *θ_y_* of r-PBS/BSGF decreases by about −15% after five processing cycles.

The increases in both *θ_w_* and *θ_y_* WCA, i.e., an increase in hydrophobicity, for r-PBS could be understood by considering that during the extrusion cycling steps, the occurrence of thermo-mechanical degradation leads to the formation of polar surface groups and as expected, water penetration is disadvantaged. Conversely, the presence of BSGF in PBS leads to a decrease in both *θ_w_* and *θ_y_* WCA (*p* < 0.0001), i.e., an increase in hydrophilicity, even more so as a function of the extrusion cycling, since the ability of BSGF to absorb water molecules overcomes the thermo-mechanical degradation of PBS matrix. Also, according to the literature, the organic particles can absorb water/humidity/moisture, and this increases the bio-composite hydrophilicity [11].

**Figure 6:**
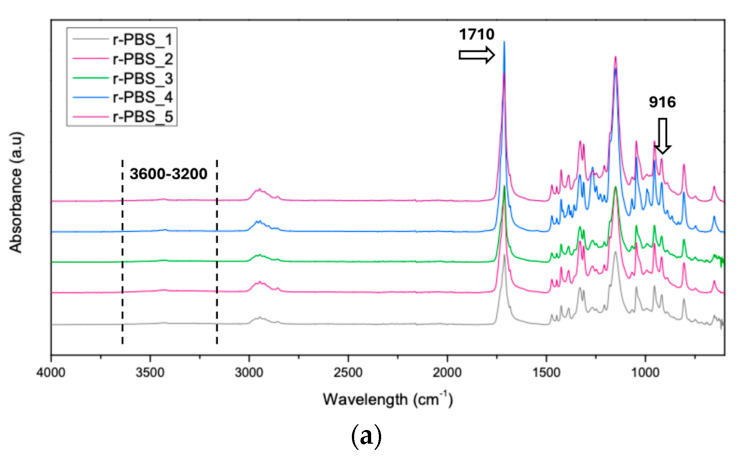

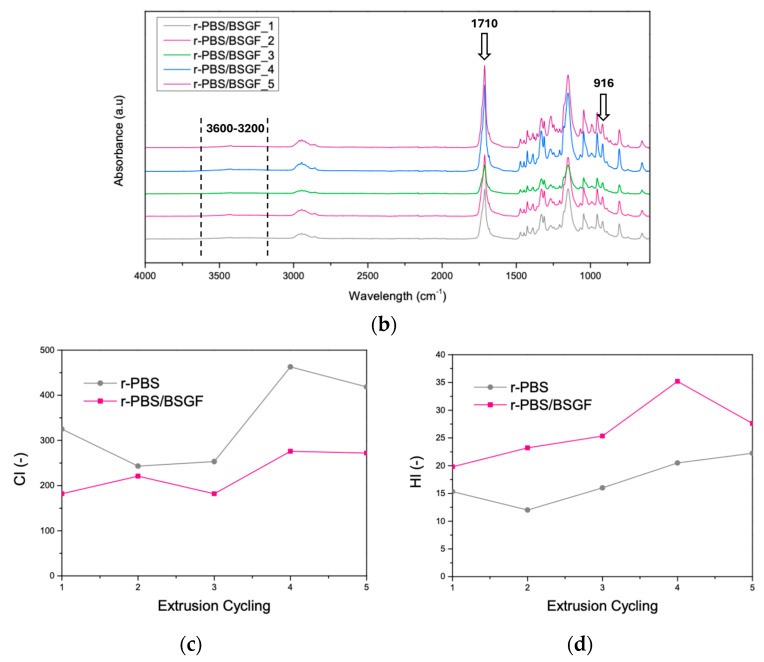
ATR-FTIR spectra of (**a**) r-PBS and (**b**) r-PBS/BSGF, and comparison of (**c**) carboxyl index and (**d**) hydroxyl index vs. extrusion cycling.

The ATR-FTIR spectra of r-PBS and r-PBS/BSGF as a function of the extrusion cycling are shown in Figure 6a,b. In addition to the spectra, the Carbonyl Index and Hydroxyl Index were calculated and are presented in Figure 6c,d. Table 1 lists the assignment of the ATR-FTIR peaks of Figure 6.

PBS is a condensation-type biopolymer characterized by hydroxyl- and carboxyl-terminated chains, with the concentration of these end groups ([OH], [COOH]) depending on the polymerisation conditions. As reported in studies on the thermal and thermo-oxidative degradation of PBS [5,15,26], the degradation mechanism mainly involves chain scission, accompanied by the formation of carboxylic acid end groups and vinyl groups. According to the literature, the formation of additional carboxylic groups reflects the increase in overall carbonyl accumulation in the 1800–1600 cm^−1^ range. Specifically for PBS, the formation of additional vinyl groups gives rise to peaks at 952 and 914 cm^−1^, which occur simultaneously with the formation of carboxylic groups, giving rise to the peak at 917 cm^−1^ attributed to the –C–OH bending in the carboxylic acid groups. The spectra of both r-PBS and r-PBS/GSGF in Figure 6a,b show a single peak at 916 cm^−1^, attributed to the bending of the intrinsic carboxylic groups that overlaps the formation of vinyl groups. It seems that the peak at 916 cm^−1^ slightly increases for both r-PBS and r-PBS/GSGF as the extrusion cycles increase.

Overall, the spectra do not show significant changes in the formation of new functional groups with the increase in extrusion cycles for both the neat and filled polymer. It is worth noting that no additional peaks are noticeable due to the presence of BSGF in PBS, probably because of the similarity in the chemical nature of PBS and BSGF. Fortunately, the presence of BSGF appears to limit the formation of carboxylic species, thereby mitigating the degradation typically associated with Norrish Type II reactions (see Figure 6c). This experimental evidence could be explained by considering that lignin, contained in BSGF, acts as an antioxidant [27]. In fact, lignin is a natural aromatic polymer whose chemical structure confers the ability to act as an antioxidant, typically protecting plant cells from free radical damage. Furthermore, lignin may also have anti-UV and antibacterial properties [28].

These reactions are responsible for the formation of carbonyl groups, which can increase brittleness and reduce melt flow, molecular weight, and mechanical strength upon exposure, an effect that is effectively suppressed by the inclusion of BSGF [29,30]. However, the inclusion of an organic component such as BSGF likely increases the hydrophilicity of the composite (as expected for their intrinsic hydrophilicity and according to the wettability test results), which may contribute to a slightly higher, though still moderate, formation of hydroperoxide and hydroxyl species (Figure 6d).

**Figure 7:**
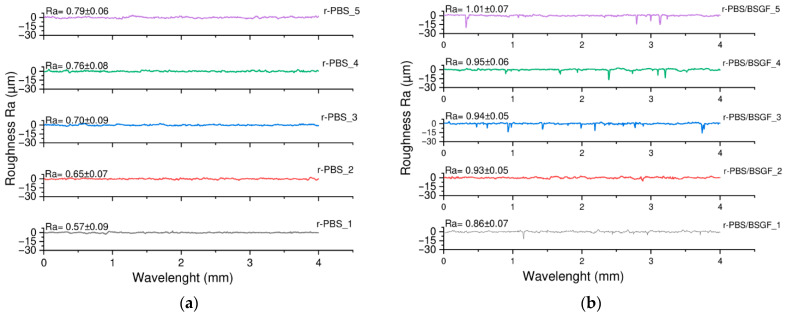
Surface roughness of (**a**) r-PBS and (**b**) r-PBS/BSGF at different extrusion cycles.

Interesting results and extremely significant results come from the surface roughness analysis of the r-PBS and r-PBS/BSGF samples investigated, see Figure 7a,b. As expected, the presence of BSGF increases the surface roughness from 0.57 μm to 0.86 μm (*p* < 0.0001). Furthermore, the roughness values for r-PBS_1 and r-PBS_5 are approximately 0.57 μm and 0.79 μm, respectively (*p* < 0.0001), indicating an increase in surface roughness of approximately 38% during the extrusion cycling. For r-PBS/BSGF_1 and r-PBS/BSGF_5, the roughness values change from 0.86 μm to 1.01 μm (*p* < 0.0001), indicating an increase of approx. 17.5%, which is significantly less than the change experienced by pure r-PBS during the extrusion cycling. The latter suggests that r-PBS/BSGF shows higher surface roughness due to the presence of BSGF, which changes less with the extrusion cycling compared to the changes for neat r-PBS.

### 3.4. Thermal Behaviour of r-PBS and r-PBS/BSGF

**Figure 8:**
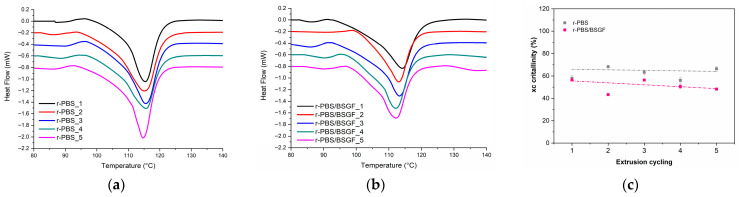
DSC first heating scans of (**a**) r-PBS and (**b**) r-PBS/BSGF and (**c**) crystallinity vs. extrusion cycling.

Figure 8a,b show the first heating scans of r-PBS and r-PBS/BSGF as a function of the temperature, and Figure 8c displays the crystallinity vs. extrusion cycles. In addition, Table 2 summarizes the values of the melting temperature (T_m_), fusion enthalpy (ΔH_m_), and calculated crystallinity (χ_c_).

It can be noticed that the values of the melting temperatures remain almost unchanged with increasing extrusion cycling. The fusion enthalpy values are lower for r-PBS/BSGF than for r-PBS, highlighting that BSGF impedes crystallinity due to good dispersion and intercalation between the biopolymer chains.

Although the crystallinity changes above and below the initial values for both r-PBS and r-PBS/BSGF, the crystallinity vs. extrusion cycles fit almost linearly, see Figure 8c. It is worth noting that the crystallinity of r-PBS increases by around 18% between the first and second cycles, after which it remains at a slightly higher level than the initial value.

For r-PBS/BSGF, the crystallinity vs. extrusion cycles shows an opposite trend. More specifically, the crystallinity decreases by around 23% between the first and second cycles. The decrease in crystallinity in the second cycle coincides with the decrease in the melt flow index (MFI) and the increase in complex viscosity, as discussed above. Therefore, the thermal behaviour reflects the transition of the biopolymer chains from a solid to a liquid state, while rheological tests (both oscillatory and MFI) are carried out in a molten state and depend on the biopolymer chains’ and BSGF’s ability to flow and become oriented during flow.

### 3.5. Mechanical Behaviour of r-PBS and r-PBS/BSGF

**Figure 9:**
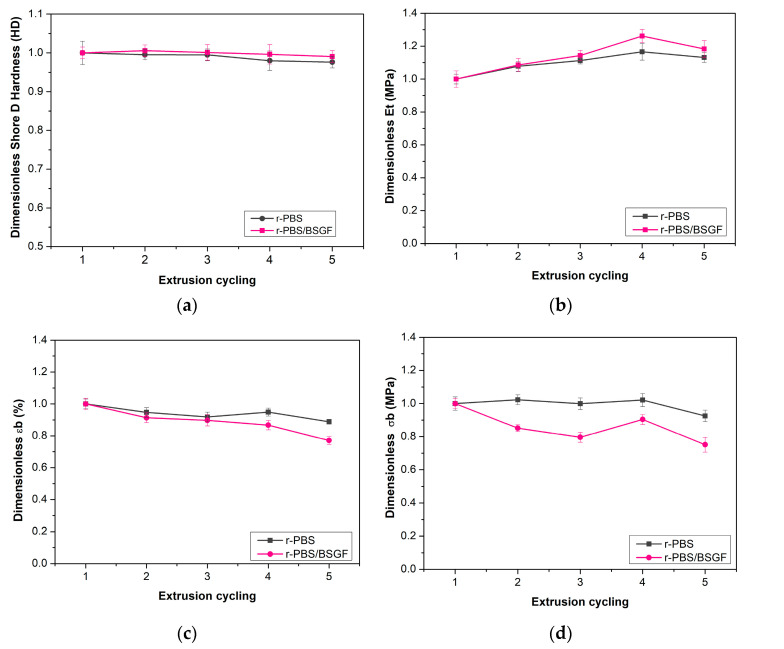
Dimensionless shore D hardness (**a**) elastic modulus, E_t_, (**b**) elongation at break, ε_b_, (**c**) and tensile strength σ_b_ (**d**) of r-PBS and r-PBS/BSGF vs. extrusion cycling.

Figure 9a–d show the dimensionless main mechanical properties, such as (a) shore D hardness, (b) Young’s (elastic) modulus, Et, (c) elongation at break, εb, and (d) tensile strength, σb. Furthermore, Table 3 reports the main values of the mechanical properties of r-PBS_1-5 and r-PBS/BSGF_1-5.

Figure 9a shows the dimensionless shore D hardness of r-PBS and r-PBS-BSGF. The decrease in shore D values from r-PBS_1 to r-PBS_5 is of −2.37% (*p* < 0.0001) while the decrease from r-PBS/BSGF_1 to r-PBS/BSGF_5 is of −0.94% (*p* = 0.0053). This result suggests that the changes in surface hardness are generally little for both r-PBS and r-PBS/BSGF with increasing extrusion cycling, as is visible from the trend of Figure 8a, but surface hardness is more stable in r-PBS-BSGF than in r-PBS during the extrusion cycling.

Figure 9b–d show the dimensionless tensile parameters of the recycled blends (Young’s modulus, elongation at break, and tensile strength) of r-PBS and r-PBS/BSGF, plotted as a function of the number of extrusion cycles. The dimensionless values of the Young modulus increase while elongation at break decreases after five extrusion cycles. These trends are more pronounced in r-PBS/BSGF compared to r-PBS, as expected, since the BSGF presence makes the bio-composite more brittle, according to the literature [14,31]. Therefore, the BSGF presence causes an increase in the elastic modulus (ca. 28–31%), and approximately a two-times reduction in the ductility, i.e., both tensile strength and elongation at break, see Table 3. As a function of the extrusion cycles, the BSGF presence does not play a negative role on the mechanical behaviour, considering that r-PBS and r-PBS/BSGF show similar trends, see Figure 9b–d.

The experimental data on the relative stiffness and tensile strength of our r-PBS_1 and r-PBS/BSGF_1 samples are comparable to the values for the B0 and B30 samples of Nnaemeka Ewurum et al. [32]. The authors of this work do not share the elongation at break data.

In detail, the Young modulus grows after five extrusion cycles of +18.3% and +13.2% in r-PBS/BSGF compared to r-PBS, respectively (*p* < 0.0001 and *p* = 0.0122, respectively). The elongation decreases after five extrusion cycles were −22.9% and −11.2% in r-PBS/BSGF compared to r-PBS, (*p* = 0.0372 and *p* = 0.0426, respectively).

The stress at break values after five extrusion cycles showed decreases of −24.9% and of −7.4%, for r-PBS/BSGF and of r-PBS, respectively, (*p* < 0.0001 and *p* = 0.0084, respectively). By observing the percentage variations, and in agreement with the literature [5], it can be confirmed that PBS can undergo up to five extrusion cycles without significant loss in tensile strength. Conversely, the bio-composite can be reprocessed profitably only up to three cycles, beyond which it shows a 20% reduction in mechanical resistance (from 1 to 0.8, see Figure 9c,d), a 10% increase in stiffness (Figure 8b), and a slight decrease in elongation at break.

The mechanical behaviour of r-PBS/BSGF is primarily influenced by the presence, dispersion and morphology achieved by BSGF. Although BSGF exerts a reinforcing action and has a positive effect on the mechanical behaviour up to three extrusion cycles, its presence in the fourth and fifth extrusion cycles makes the bio-composites significantly stiffer, which limits their recyclability. r-PBS appears to demonstrate good recyclability potential with minimal changes to its mechanical properties.

## 4. Conclusions

In this work, the rheological, physical, morphological, and mechanical properties of PBS and PBS-BSGF bio-composite were analyzed during five cycles of extruder reprocessing.

The results obtained showed that the presence of BSGF has a positive effect on the PBS resistance to degradation during the extrusion cycles, stabilizing the molten state during reprocessing steps and limiting the formation of carboxylic groups. Interestingly, according to rheological and morphological analyses, two extrusion cycles are necessary to improve the dispersion of the BSGF inside the PBS biopolymer matrix.

The hydrophilic nature of BSGF means that they absorb more water; therefore, BSGF makes the bio-composite more hydrophilic compared to pure PBS, which, during the processing cycles, is made less wettable. The WCA of r-PBS_1 and r-PBS_5 is ca. 84° and ca. 90°, respectively, while for r-PBS/BSGF_1 and _5 it decreases from 75° to 64°.

The PBS/BSGF is rougher than PBS due to the presence of the filler; however, the roughness of PBS/BSGF changes less during processing cycles than the PBS surface. Overall, the surface hardness for both r-PBS and r-PBS/BSGF remains quite unchanged during the five extrusion cycles. Furthermore, both PBS and PBS/BSGF increase their stiffness and decrease their deformability and tensile strength during the five extrusion cycles due to progressive breaking. This effect is more pronounced in the bio-composite than in the bioplastic.

Therefore, from the point of view of mechanical behaviour (surface hardness, Young’s modulus, elongation at break, and tensile strength), PBS is more recyclable than the bio-composite since it can withstand five extrusion cycles. Instead, bio-composites can be recycled profitably only two more times (up to cycle three).

## Figures and Tables

**Table 1:** Assignment of ATR-FTIR peaks of neat PBS [5,24,25,26].

Assigned Peak (cm^−1^)	Chemical Shift/Groups
3200–3600	hydroperoxide and hydroxyl species
2963, 2925, 2859	–CH_2_ and –CH stretching
1710	C=O stretching
1159	C-O stretching
1333	symmetric C–O stretching
1046	O(CH_2_)_4_O vibration
952, 914	Vinyl groups [5,25]
917	–C–OH bending in the carboxylic acid groups [24]
955 (*)	C–O symmetric stretching [5,25]
806 (*)	–CH_2_ in OC(CH_2_)_2_CO in-plane bending [5,25]
656 (*)	COO bending [5,25]

Note: (*) These three peaks (if simultaneously presented) can be used as indicative peaks to discriminate PBS from other bioplastics [26].

**Table 2:** DSC data related to the first heating.

Sample	T_m_ (°C)	ΔH_m_ (J/g)	χ_c_ (%)	Sample	T_m_ (°C)	ΔH_m_ (J/g)	χ_c_ (%)
**r-PBS_1**	115.3 ± 3.6	63.9 ± 2.4	57.9 ± 2.6	**r-PBS/BSGF_1**	114.1 ± 1.8	62.1 ± 2.6	56.3 ± 0.5
**r-PBS_2**	115.1 ± 4.4	75.3 ± 3.3	68.3 ± 1.1	**r-PBS/BSGF_2**	113.0 ± 3.7	47.8 ± 2.5	43.3 ± 1.2
**r-PBS_3**	115.5 ± 5.3	69.8 ± 2.3	63.3 ± 2.2	**r-PBS/BSGF_3**	113.2 ± 2.0	62.2 ± 3.0	56.4 ± 0.9
**r-PBS_4**	115.6 ± 3.7	61.8 ± 1.7	56.0 ± 2.7	**r-PBS/BSGF_4**	112.1 ± 4.7	55.7 ± 2.2	50.5 ± 1.8
**r-PBS_5**	114.7 ± 4.8	73.2 ± 3.1	66.4 ± 2.1	**r-PBS/BSGF_5**	112.3 ± 2.7	53.2 ± 2.0	48.3 ± 0.7

**Table 3:** Main mechanical properties, i.e., shore D hardness, Young’s (elastic) modulus, Et, elongation at break, εb, and tensile strength, σb, of r-PBS and r-PBS/BSGF at different extrusion cycles.

Sample	Shore D [HD]	Et [MPa]	εb [%]	σb [MPa]	Sample	Shore D [HD]	Et [MPa]	εb [%]	σb [MPa]
**r-PBS_1**	64.86 ± 0.60	778.8 ± 52.9	9.9 ± 1.0	32.9 ± 1.7	**r-PBS/BSGF_1**	63.58 ± 0.30	1090.3 ± 91.8	4.8 ± 0.6	18.1 ± 1.1
**r-PBS_2**	64.56 ± 0.27	839.0 ± 57.9	9.4 ± 1.2	33.7 ± 1.5	**r-PBS/BSGF_2**	63.94 ± 0.32	1184.2 ± 61.5	4.4 ± 0.6	15.4 ± 1.8
**r-PBS_3**	64.54 ± 0.31	865.7 ± 41.5	9.1 ± 1.2	32.9 ± 1.6	**r-PBS/BSGF_3**	63.66 ± 0.37	1246.3 ± 58.8	4.3 ± 0.6	14.4 ± 1.5
**r-PBS_4**	63.56 ± 0.47	907.3 ± 71.8	9.4 ± 0.9	33.6 ± 1.6	**r-PBS/BSGF_4**	63.36 ± 0.34	1375.5 ± 66.3	4.2 ± 0.5	16.3 ± 1.0
**r-PBS_5**	63.32 ± 0.30	880.7 ± 55.7	8.8 ± 1.1	30.5 ± 1.6	**r-PBS/BSGF_5**	62.98 ± 0.33	1290.3 ± 85.2	3.7 ± 0.2	13.6 ± 1.4

## Data Availability

The data sets generated during and/or analyzed during this study are available from the corresponding author on reasonable request.
